# Caspase-1 Dependent IL-1β Secretion and Antigen-Specific T-Cell Activation by the Novel Adjuvant, PCEP

**DOI:** 10.3390/vaccines2030500

**Published:** 2014-06-26

**Authors:** Sunita Awate, Nelson F. Eng, Volker Gerdts, Lorne A. Babiuk, George Mutwiri

**Affiliations:** 1 Vaccinology and Immunotherapeutics program, School of Public Health, 107 Wiggins Road, University of Saskatchewan, Saskatoon, SK S7N 5E5, Canada; E-Mail: sunita.awate@usask.ca; 2 Vaccine and Infectious Disease Organization-International Vaccine Centre, 120 Veterinary Road, University of Saskatchewan, Saskatoon, SK S7N 5E3, Canada; E-Mail: volker.gerdts@usask.ca (V.G.); 3 Department of Veterinary Microbiology, Western College of Veterinary Medicine, University of Saskatchewan, Saskatoon, SK S7N 5B4, Canada; 4 University of Alberta, 3-7 University Hall, Edmonton, AB T6G 2J9, Canada; E-Mail: lorne.babiuk@ualberta.ca

**Keywords:** adjuvants, polyphosphazenes, inflammasomes, IL-1β, T-cells

## Abstract

The potent adjuvant activity of the novel adjuvant, poly[di(sodiumcarboxylatoethylphenoxy)phosphazene] (PCEP), with various antigens has been reported previously. However, very little is known about its mechanisms of action. We have recently reported that intramuscular injection of PCEP induces NLRP3, an inflammasome receptor gene, and inflammatory cytokines, including IL-1β and IL-18, in mouse muscle tissue. Caspase-1 is required for the processing of pro-forms of IL-1β and IL-18 into mature forms and is a critical constituent of the NLRP3 inflammasome. Hence, in the present study, we investigated the role of caspase-1 in the secretion of IL-1β and IL-18 in PCEP-stimulated splenic dendritic cells (DCs). Caspase inhibitor YVAD-fmk-treated splenic DCs showed significantly reduced IL-1β and IL-18 secretion in response to PCEP stimulation. Further, PCEP had no effect on the expression of MHC class II or co-stimulatory molecules, CD86 and CD40, suggesting that PCEP does not induce DC maturation. However, PCEP directly activated B-cells to induce significant production of IgM. In addition, PCEP+ovalbumin (OVA) immunized mice showed significantly increased production of antigen-specific IFN-γ by CD4^+^ and CD8^+^ T-cells. We conclude that PCEP activates innate immunity, leading to increased antigen-specific T-cell responses.

## 1. Introduction

Modern vaccines with highly purified antigens require the addition of adjuvants to enhance the immune responses. Although the mechanisms of adjuvants are largely unknown, a few mechanisms have been proposed, including, depot formation, an increase in cytokine and chemokine production, immune cell recruitment, enhanced antigen uptake and presentation by antigen presenting cells (APCs) [1,2,3]. Generally, adjuvants utilize a combination of these various mechanisms to promote antigen-specific immune responses.

Various studies have shown that TLR-dependent signaling is not required by alum and MF59 to induce antigen-specific antibody responses [4,5]. However, antigen-specific T-cell responses induced by alum depend on myeloid differentiation primary response gene 88 (MyD88) and uric acid [6]. Uric acid is released as a result of cell damage and necrosis induced by alum at the injection site, which act as danger signals for the activation of the NOD-like receptor family, pyrin-domain-containing 3 (NLRP3). Activation of the NLRP3 inflammasome, an intra-cytoplasmic multi-protein complex, induces caspase-1 activation, which, in turn, cleaves pro-forms of IL-1β, IL-18 and IL-33 to their bioactive forms [7]. Alum-induced secretion of IL-1β and IL-18 was shown to be caspase-1 dependent [8]. However, the role of NLRP3 in the adjuvant activity of alum *in vivo* is not clear.

Polyphosphazenes are a novel class of adjuvants that have been shown to be effective as parenteral and mucosal adjuvants in small, as well as large animals [9,10]. In particular, the new generation polyphosphazene polyelectrolyte, poly[di(sodiumcarboxylatoethylphenoxy)phosphazene] (PCEP), promotes enhanced and long-lasting immune responses with a variety of viral and bacterial antigens [9,11,12,13,14]. However, the mechanisms by which PCEP induces higher immune responses are poorly understood. We have previously shown that PCEP stimulates the production of innate cytokines in mouse splenocytes and induces the production of various cytokines and chemokines at the site of injection [12,15]. In addition, PCEP enhanced the expression of the NLRP3 gene and induced the local production of pro-inflammatory cytokines IL-1β and IL-18 [15]. In the present study, we investigated the role of caspase-1 in PCEP-mediated pro-inflammatory cytokine production, the potential of PCEP to directly activate DCs and the capacity of PCEP to induce antigen-specific cellular responses in mice.

## 2. Experimental

### 2.1. Animals

Four- to six-week-old female BALB/c mice purchased from Charles River Laboratories (North Franklin, CT, USA) were used in all of the experiments. The animal experiments were approved by the University of Saskatchewan’s Animal Research Ethics Board and adhered to the Canadian Council on Animal Care guidelines for the humane use of animals.

### 2.2. Adjuvants

PCEP was synthesized by Idaho National Laboratories (Idaho Falls, ID, USA) using methods described previously [16,17], and prior to use, its endotoxin levels were determined to be less than 0.034 ng/mL, as assessed by the Limulus Amebocyte Lysate assay (Biowhittaker, Walkersville, MD, USA). PCEP was dissolved in Dulbecco’s phosphate buffered saline (PBS) (Sigma-Aldrich, St. Louis, MO, USA) by gentle agitation for 36 h at room temperature (RT). Imject alum (Thermo Fisher Scientific, Waltham, IL, USA) used in these experiments was a mixture of alum and magnesium hydroxide (40 mg/mL). Lipopolysaccharide (LPS) was purchased from InvivoGen (San Diego, CA, USA).

### 2.3. Isolation and Culture of Splenic-Derived DCs

Spleen cells were disrupted by injecting collagenase D (Roche Diagnostics, Basel, Switzerland) solution (2 mg/mL in 10 mM HEPES-NaOH pH 7.4, 150 mM NaCl, 5 mM KCl, 1 mM MgCl_2_, 1.8 mM CaCl_2_) into the spleen. Disrupted spleen tissues were incubated at 37 °C for 30 min. The spleen tissues were teased with a syringe plunger through the nylon mesh to obtain the cell suspension. Total DCs (conventional and plasmacytoid DCs) from the mouse spleen cell suspension were positively selected using Pan DC microbeads (Miltenyi Biotec, Bergisch Gladbach, Germany), according to the manufacturer’s instructions. Isolated, enriched DCs were stained with CD11c-PE and Anti-mPDCA-1-APC (both from eBiosciences, San Diego, CA, USA) to check for purity using flow cytometry (the purity of the isolated enriched splenic DCs was found to be >80%).

Magnetic-activated cell sorting (MACS) isolated, enriched splenic DCs were cultured (1 × 10^6^ cells/well) with media, PCEP (50 μg/mL), alum (0.5 mg/mL), LPS (0.1 μg/mL), PCEP+LPS or alum+LPS. The dosage of PCEP was selected based on previous experiments [12]. In some experiments, enriched DCs were incubated with caspase-inhibitor YVAD-fmk (R&D Systems, Minneapolis, MN, USA) along with vaccine adjuvants. After 12 h of stimulation, culture supernatants and cells were collected for cytokine measurement and immunoblot, respectively. The IL-1β concentration was assayed in culture supernatants using the DuoSet ELISA development system (R&D Systems), following the manufacturer’s instructions, and IL-18 was measured as described earlier [15].

### 2.4. Immunoblotting

After 12 h of culture, enriched splenic DCs were lysed with RIPA buffer (0.5 M Tris (pH 8.0), 0.15 M NaCl, 0.1% SDS, 1% NP-40, 1% deoxycholic acid) containing protease inhibitors (1× Complete Protease Inhibitor; Roche Diagnostics). Thirty micrograms of total protein from each lysate were subjected to 12.5% SDS-polyacrylamide gel electrophoresis and then transferred to a nitrocellulose membrane (Bio-Rad, Hercules, California, USA). The membrane was probed for pro-IL-1β (Santa Cruz Biotechnology, CA, USA) diluted 1:40 and procaspase-1 (Santa Cruz Biotechnology) diluted 1:200, followed by incubation with infrared dye (IRDye) secondary antibodies (LI-COR, Lincoln, NE, USA) diluted 1:5000. Finally, infrared signals of immunoblots were detected by an Odyssey infrared imager (LI-COR). Immunoblotting for β-actin (Sigma-Aldrich) served as a loading control.

### 2.5. Generation and Culture of BMDCs

Bone marrow (BM) cells were cultured in complete RPMI media (containing 10% fetal calf serum (FCS), HEPES, non-essential amino acids, sodium pyruvate, antibiotic/antimycotic (all from Gibco, Grand Island, NY, USA) and β-mercaptoethanol (Sigma-Aldrich)) supplemented with 100 ng/mL recombinant mouse Fms-related tyrosine kinase 3 ligand (Flt3L; PeproTech, Rocky Hill, NJ, USA) at 1 × 10^6^ cells/mL in a 6-well plate (Corning, Corning, NY, USA) for 7 days. On the 7th day, immature DCs (iDCs) were harvested and again resuspended in complete RPMI supplemented with murine Flt3L and cultured for 24 h with media, PCEP (50 μg/mL) or LPS (100 ng/mL) at 1 × 10^6^ cells/mL in a 24-well plate. After 24 h, bone marrow-derived dendritic cells (BMDCs) were harvested and stained with MHC class II, CD86 and CD40 antibodies (all from eBiosciences).

### 2.6. Splenic B-Cell Isolation

Spleens were aseptically removed from naive/untreated mice and placed in ice-cold collagenase D solution. Spleen cells were disrupted by injecting collagenase D solution into the spleen with a syringe, later cut into smaller pieces and incubated at 37 °C for 30 min. After incubation, spleen tissues were teased with a syringe plunger through the nylon mesh. The cell suspension obtained was resuspended with autoMACS rinsing buffer with 0.5% BSA (Miltenyi Biotec). B-cells were positively selected from the mouse spleen cell suspension using CD45R (B220) microbeads (Miltenyi Biotec), according to the manufacturer’s instructions. Isolated, enriched B-cells were stained with CD19-FITC (eBiosciences) to check for purity using flow cytometry (the purity of enriched B-cells was found to be >80%).

### 2.7. B-Cell Culture and Proliferation Assay

MACS isolated, enriched splenic B-cells (2 × 10^6^ cells/well) were cultured in the presence of media, PCEP (10 μg/mL) or LPS (0.1 μg/mL) in a humidified incubator containing 5% CO_2_ at 37 °C. Culture supernatants were collected after 48 h for IL-6 (DuoSet ELISA development system; R&D Systems) and immunoglobulin M (IgM) (Ready-SET-Go kit; eBiosciences) quantification. For the proliferation assay, a triplicate wells of enriched naive B-cells (2 × 10^5^ cells/well) were cultured in the presence of medium, PCEP (5 μg/mL, 10 μg/mL and 25 μg/mL) and LPS (0.1 μg/mL) into 96-well round bottomed plates for 5 days. The cells were pulsed with 0.4 μCi/well of titrated thymidine (American Radiolabeled Chemicals (ARC), St. Louis, MO, USA) during the last 18 h of culture. Thymidine incorporation was measured using a liquid scintillation counter (PerkinElmer, Waltham, MA, USA). Results were expressed as the stimulation index. The stimulation index represents the ratio of counts per minute (cpm) obtained in the stimulated cultures to cpm obtained in controls (media). A stimulation index of ≥3 indicates a positive proliferation response.

### 2.8. Immunization of Mice

Mice were divided into groups and immunized intramuscularly (i.m.) with 25 μL each of either PBS as a control, 10 µg ovalbumin (OVA) (Hyglos GmbH, Bavaria, Germany) or 50 µg of PCEP co-delivered with 10 µg OVA. The endotoxin concentration in OVA used in immunization studies was <1 EU/mg. Half of the mice in each group were euthanized 9 days after immunization to collect spleens. The remaining mice were given a secondary immunization on Day 14 and euthanized 21 days after the first immunization to collect spleens.

### 2.9. Intracellular IFN-γ Staining

Spleens were digested with collagenase solution to get a single cell suspension. To investigate IFN-γ production, splenocytes (1 × 10^6^ cells/well) were cultured in 96-well round bottomed culture plates and restimulated with 10 μg/mL OVA and incubated at 37 °C and 5% CO_2_ in a humidified incubator. Intracellular staining for IFN-γ was performed after 12 h of incubation. Cells were fixed with 4% paraformaldehyde (RICCA chemicals, Arlington, TX, USA) and stained with CD4 and CD8a T-cell markers (eBiosciences). Subsequently, cells were permeabilized with cytofix/cytoperm (BD Biosciences, San Jose, CA, USA) and stained for intracellular IFN-γ (BD Biosciences). Enumeration of IFN-γ responses by CD4^+^ and CD8a^+^ T-cells was done by flow cytometric analysis.

### 2.10. Statistical Analysis

Statistical analysis was carried out using GraphPad Prism 5 software (GraphPad Software, San Diego, CA, USA). The differences between groups were analyzed by one-way ANOVA, and statistical significance between the treatments was compared with Dunn’s and Tukey’s multiple comparison test; *** *p* < 0.0001, ** *p* < 0.001, * *p* < 0.05.

## 3. Results

### 3.1. PCEP Induces Robust Secretion of IL-1β and IL-18 in Splenic DCs

**Figure 1:**
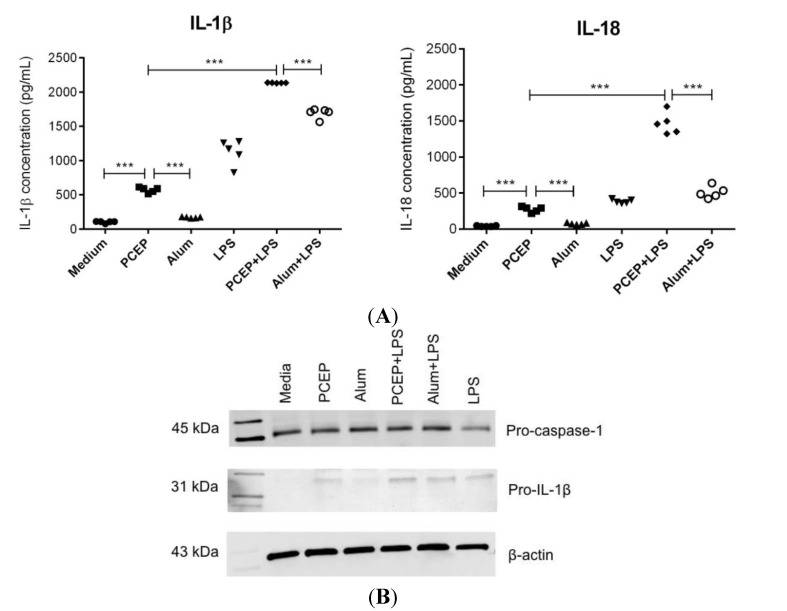
Poly[di(sodiumcarboxylatoethylphenoxy)phosphazene] (PCEP) induces robust secretion of IL-1β and IL-18 in splenic dendritic cells (DCs). Enriched splenic DCs from BALB/c mice were incubated for 12 h with media, PCEP (50 μg/mL), alum (40 mg/mL), lipopolysaccharide (LPS) (0.1 μg/mL), PCEP+LPS or alum+LPS. Supernatants were collected for measuring IL-1β and IL-18 by ELISA, and the cell extracts were analyzed for pro-IL-1β by western blotting. (**A**) Secretion of IL-1β and IL-18 in enriched splenic DCs; (**B**) pro-caspase-1 (45 kDa), pro-IL-1β (31 kDa) and β-actin (43 kDa) detection in cell lysates by western blot analysis. Data was analyzed by one-way ANOVA, and the comparisons between the treatments were done by Tukey’s multiple comparison test; *** *p* < 0.0001.

Stimulation of enriched splenic DCs with PCEP induced significantly higher IL-1β and IL-18 secretion relative to media control and alum. In addition, stimulation with PCEP in the presence of LPS triggered significantly higher secretion of IL-1β and IL-18 compared to PCEP or LPS alone (Figure 1A). Further, in the presence of LPS, PCEP induced significantly higher secretion of IL-1β and IL-18 compared to the alum+LPS combination (Figure 1A).

Since PCEP alone was able to induce significant IL-1β secretion, we assessed the induction of pro-IL-1β and pro-caspase-1 by PCEP. Enriched splenic DCs were stimulated with PCEP or alum in the presence or absence of LPS for 12 h, and cell extracts were analyzed for pro-IL-1β and pro-caspase-1 by western blot. We observed that PCEP alone induced the intracellular production of pro-IL-1β in enriched splenic DCs (Figure 1B). However, pro-IL-1β induction was not as strong as the induction by the PCEP+LPS combination. Pro-caspase-1 was produced in all of the enriched splenic DC treatments, including media control, whereas pro-IL-1β was not present before stimulation. β-actin was used as a positive control in all western blot experiments.

### 3.2. PCEP Mediated IL-1β Secretion is Caspase-1 Dependent

**Figure 2:**
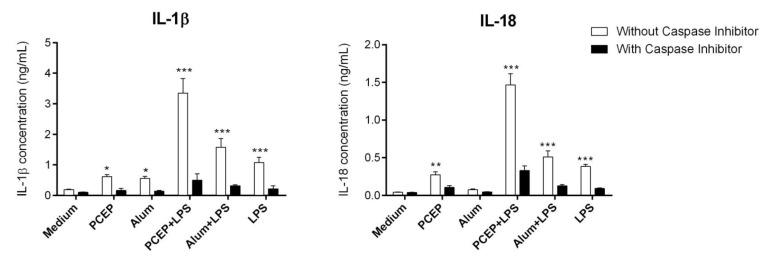
The role of caspase-1 in PCEP-stimulated IL-1β and IL-18 secretion. Enriched splenic dendritic cells (DCs) were treated with or without the caspase-1 inhibitor (CI) YVAD-fmk (40 μM) and then incubated with media, PCEP (50 μg/mL), alum (40 mg/mL), lipopolysaccharide (LPS) (0.1 μg/mL), PCEP+LPS or alum+LPS. Supernatants were collected after 12 h of stimulation and were analyzed for IL-1β and IL-18 by ELISA. Data were analyzed by one-way ANOVA, and the comparisons between the treatments were done by Tukey’s multiple comparison test; *** *p* < 0.0001, ** *p* < 0.001, * *p* < 0.05.

Caspase-1 is a critical component of the NLRP3 inflammasome, which cleaves the pro-form of IL-1β and IL-18 into mature forms. Hence, we examined the role of caspase-1 in the secretion of IL-1β and IL-18 by splenic DCs. Enriched splenic DCs were treated with or without the caspase-1 inhibitor (CI) YVAD-fmk and then stimulated with media, PCEP, alum, LPS, PCEP+LPS or alum+LPS for 12 h. The secretion of IL-1β and IL-18 was analyzed in culture supernatants. Pre-treatment with YVAD-fmk significantly inhibited IL-1β and IL-18 secretion (Figure 2). The most significant reduction in IL-1β and IL-18 secretion was observed in YVAD-fmk-treated enriched DCs that were stimulated with PCEP+LPS (Figure 2). The same was observed with alum+LPS-treated enriched splenic DCs. These results suggest that PCEP- and alum-mediated secretion of IL-1β and IL-18 in splenic DCs was caspase-1 dependent.

### 3.3. PCEP Does Not Induce MHC Class II and Co-Stimulatory Molecules Expression in Vitro

**Figure 3:**
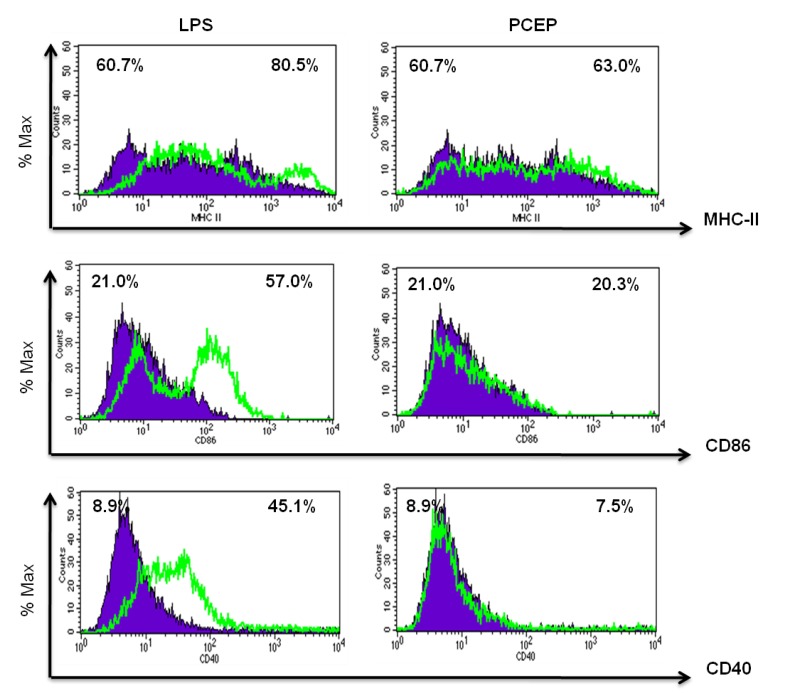
PCEP does not induce MHC class II and co-stimulatory molecules expression *in vitro*. Bone marrow-derived dendritic cells (BMDCs) (1 × 10^6^ cells/mL) were incubated with media, PCEP (50 μg/mL) or lipopolysaccharide (LPS) (100 ng/mL) for 24 h. Cells were stained with MHC class II, CD 86 and CD40 antibodies and analyzed by flow cytometry. The overlay histograms show the % of maximum cells positive for MHC class II, CD86 or CD40 in PCEP- and LPS-treated BMDCs. The blue shaded area represents media control, and the green overlay line represents LPS- and PCEP-treated BMDCs.1.

PCEP did not induce significant MHC class II and co-stimulatory molecules CD86 and CD40 expression in BMDCs compared to negative controls (Figure 3). In contrast, MHC class II, CD86 and CD40 molecules were highly expressed in LPS-treated BMDCs (Figure 3).

### 3.4. PCEP Induces Direct Activation of Naive B-Cells in Vitro

**Figure 4:**
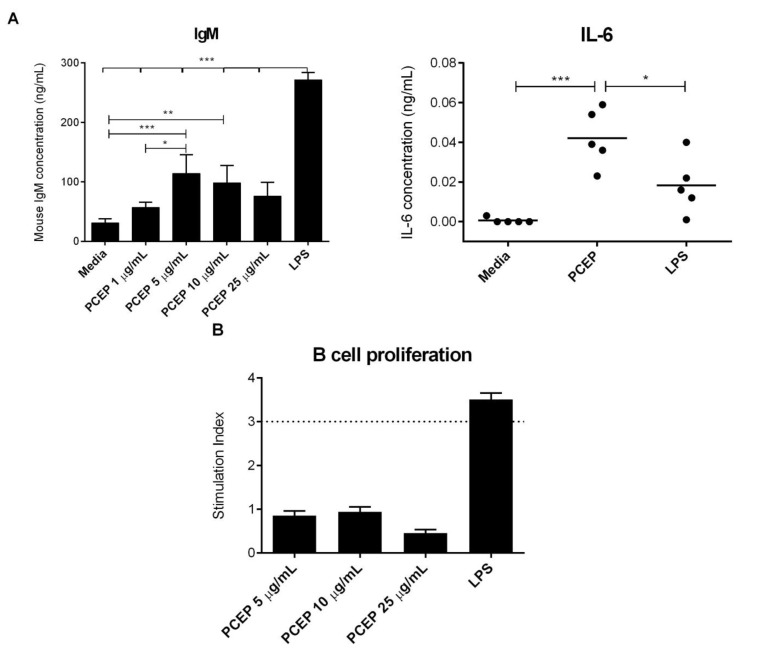
PCEP induces direct activation of naive B-cells *in vitro*. (**A**) Enriched splenic CD19^+^ B-cells (2 × 10^6^) were cultured in the presence of media, PCEP (10 μg/mL) or lipopolysaccharide (LPS) (0.1 μg/mL), and culture supernatants were collected after 48 h for quantification of IL-6 and IgM by ELISA; (**B**) enriched naive B-cells (2 × 10^5^ cells/well) were co-cultured with irradiated splenocytes in the presence of medium, PCEP (5 μg/mL, 10 μg/mL and 25 μg/mL) and LPS (0.1 μg/mL) for five days. PCEP-specific B-cell proliferative responses were measured by 3H-thymidine incorporation. Results are expressed as stimulation indexes (counts per minute (cpm) in the stimulated cultures/cpm in the controls). Tests were carried out in triplicate. A stimulation index of ≥3 was considered positive for proliferative responses (above dashed lines). All of the ELISA data were statistically analyzed by one-way ANOVA, and the differences between the treatments were compared by Tukey’s multiple-comparison test; *** *p* < 0.0001, ** *p* < 0.001, * *p* < 0.05.

MACS isolated, enriched CD19^+^ B-cells were cultured in the presence of PCEP, and the culture supernatants were analyzed for cytokine and IgM responses. PCEP stimulated significant production of IgM in a dose-dependent manner, with the highest production when used at 5 µg/mL, suggesting the direct activation of enriched naive B-cells (Figure 4A). In addition, PCEP induced significant production of IL-6; however, the amounts of IL-6 produced was low (Figure 4A). Furthermore, PCEP did not induce IL-10 and IL-12 production by enriched naive B-cells (data not shown). To determine whether PCEP can induce B-cell proliferation directly, we performed *in vitro* B-cell proliferation assays using LPS as a positive control. PCEP did not induce positive proliferation responses in enriched B-cells (Figure 4B) at any concentrations. The data suggest that although PCEP induces direct activation of naive B-cells, but does not induce B-cell proliferative responses.

### 3.5. PCEP Induces Increased IFN-γ Production in CD4^+^ and CD8^+^ T-Cells

**Figure 5:**
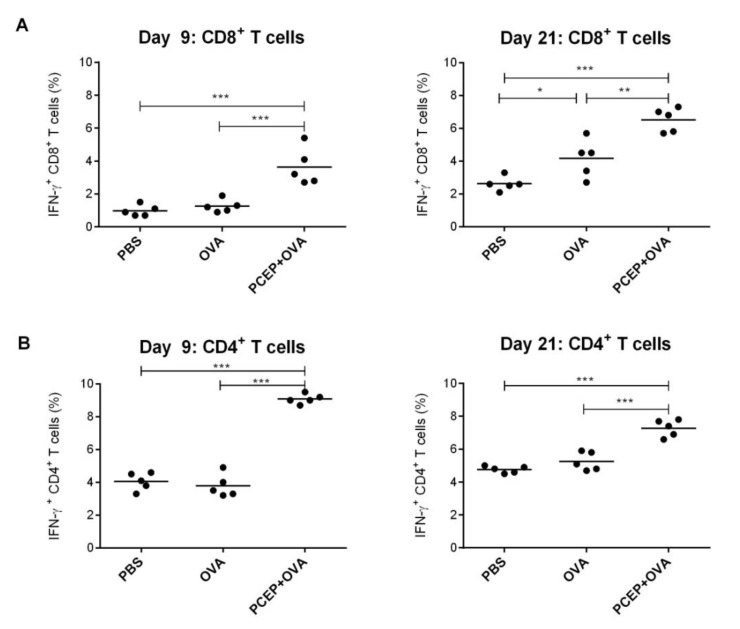
PCEP induces antigen-specific IFN-γ production in CD4^+^ and CD8^+^ T-cells. BALB/c mice were immunized i.m. with 25 μL each of either phosphate-buffered saline (PBS) as a control, 10 µg ovalbumin (OVA) or 50 μg of PCEP co-delivered with 10 μg OVA. Booster immunization was given on Day 14 to half of the mice in each group. Mice were euthanized on Days 9 and 21 after the first immunization to collect spleens. Splenocytes (1 × 10^6^ cells) were restimulated with OVA (10 μg/mL) in culture for 12 h. Intracellular production of IFN-γ by CD8^+^ (**A**) and CD4^+^ (**B**) T-cells was analyzed by flow cytometry. Statistical analysis was done by one-way ANOVA, and the differences between the treatments were compared by Tukey’s multiple-comparison test; *** *p* < 0.0001, ** *p* < 0.001, * *p* < 0.05.

To evaluate antigen-specific T-cell responses induced by PCEP, BALB/c mice were immunized with PCEP co-delivered with OVA. As shown in Figure 5, intracellular IFN-γ production was increased in mice immunized with PCEP+OVA compared with OVA alone. The frequencies of IFN-γ^+^ CD8^+^ T-cells on Day 9 (3.6% *vs.* 1.3%) and Day 21 (6.5% *vs.* 4.2%) were significantly higher in mice immunized with PCEP + OVA than in mice immunized with OVA alone (Figure 5A). Similarly, the frequencies of IFN-γ^+^ CD4^+^ T-cells on Day 9 (9.0% *vs.* 3.8%) and Day 21 (7.3% *vs.* 5.3%) were significantly higher in mice immunized with PCEP + OVA than in mice immunized with OVA alone (Figure 5B). These results indicate that PCEP induces antigen-specific activation of CD8^+^ and CD4^+^ T-cells.

## 4. Discussion

Polyphosphazenes have shown great potential as vaccine adjuvants. However, the mechanisms by which they induce these immune responses are largely unknown. Hence, the present investigations were done to understand the mechanisms of action of PCEP*.* Stimulation of enriched splenic DCs with PCEP led to the secretion of pro-inflammatory cytokines IL-1β and IL-18 in a caspase-1-dependent manner. PCEP did not induce MHC class II and co-stimulatory molecules expression in DCs. However, we observed that PCEP directly activates enriched B-cells. In addition, PCEP does induce antigen-specific IFN-γ in both CD8^+^ T-cells and CD4^+^ T-cells.

The secretion of pro-inflammatory cytokine IL-1β requires two signals: (1) synthesis of pro-IL-1β mediated via TLR agonists; and (2) activation of the inflammasome complex (NLRP3) leading to activation of caspase-1, which, in turn, cleaves pro-IL-1β, allowing the release of mature IL-1β [18]. Caspase-1-dependent secretion of pro-inflammatory cytokines IL-1β and IL-18 by alum adjuvant-stimulated DCs was first reported by Li and his colleagues [8]. Later, it was shown that the activation of NLRP3 is a pre-requisite for the alum-induced IL-1β and IL-18 secretion [19,20,21,22]. Previously, we have shown that PCEP induced the local production of various cytokines and chemokines at the site of injection [15]. In particular, PCEP induced significant production of pro-inflammatory cytokines IL-1β and IL-18 in muscle tissue. PCEP also upregulated the expression of the NLRP3 gene at the injection site [15]. In the present study, we found that PCEP-induced IL-1β and IL-18 secretion was caspase-1 dependent, strongly suggesting the involvement of NLRP3 in PCEP adjuvant activity. In addition, PCEP alone was able to induce pro-IL-1β in enriched splenic DCs, and the production of pro-IL-1β was increased when co-induced with LPS. Similarly, pre-priming with LPS was required for alum-induced secretion of IL-1β and IL-18 [19,22].

However, the role of inflammasomes in the alum adjuvant activity is disputable. Various studies using NLRP3-deficient mice showed contradictory immune responses after immunization with alum [19,20,21,22]. The contradictory *in vivo* studies have been ascribed to different immunization protocols and the types of alum and mouse strains used in the studies. However, there was a reduction in the influx of inflammatory cells in NLRP3-deficient mice, indicating the role of inflammasomes in activating innate immunity, although its role in the activation of adaptive immunity remains unclear. In regard to other adjuvants, NLRP3 is dispensable for the adjuvant activity of MF59, except for an adaptor molecule, ASC (apoptosis-associated speck-like protein containing a caspase recruitment domain), which is required for the assembly of the inflammasome [5,23]. Likewise, monophosphoryl lipid A (MPL) failed to induce caspase-1-dependent secretion of IL-1β and IL-18 [24]. PCEP is a potent activator of numerous cytokines, including IL-1β and IL-18, at the injection site [15], suggesting that although PCEP-induced IL-1β and IL-18 secretion was caspase-1 dependent, it is unlikely that the adjuvant activity of PCEP would be dependent only on IL-1β or NLRP3.

Previously, we have shown that PCEP upregulates the production of various chemokines, including CCL2, CCL4, CCL12 and CXCL10, at the injection site [15]. Due to chemotactic potential of these chemokines, we observed the increased recruitment of various myeloid and lymphoid cells to the PCEP-injected muscle tissue [25]. DCs were the first to be recruited and increased in the highest numbers in the draining lymph nodes within 3 h post-injection of PCEP [25]. Given the fact that DCs are one of the most important professional APCs involved in antigen processing and presentation to induce adaptive immune responses, it will be important to evaluate the potential of PCEP to activate DCs. Splenic DCs were partially matured and showed increased expression of MHC class II molecules (data not shown), and therefore, we used BMDCs for the DC activation studies. PCEP failed to induce the direct activation of DCs *in vitro*. Alum and MF59-treated monocytes, macrophages and granulocytes showed increased surface expression of MHC class II and co-stimulatory molecules CD83 and CD86. However, alum and MF59 failed to induce the direct activation of human DCs [26,27]. Alum does not enter DCs directly, but rather interacts with DC membrane lipids to delivers the antigen. This activates DCs, induces the expression of co-stimulatory molecules (CD80 and CD86) and adhesion molecules (intracellular adhesion molecule-1 (ICAM-1)) leading to strong contact with CD4^+^ T-cells, which subsequently leads to the B-cell response [28]. Although PCEP failed to induce DC maturation, we observed that B-cells were directly activated by PCEP *in vitro*. Further, PCEP has been shown to induce potent antigen-specific antibody responses with various bacterial and viral antigens [9,10,11,12,16,29,30,31].

Mice primed with OVA plus alum have been shown to induce CD8^+^ T-cell responses via cross-presentation by specialized CD8α^+^ DCs [32]. Further, alum plus OVA-primed CD8 T-cells differentiated into IFN-γ producing cells, whereas CD4 T-cells differentiated into IL-4-producing cells [32]. In comparison with alum, PCEP promotes antigen-specific mixed Th1 and Th2 type immune responses [16]. Immunization of mice with OVA plus PCEP induced antigen-specific IFN-γ production by both splenic CD8^+^ and CD4^+^ T-cells.

Overall, we have shown that PCEP-mediated secretion of pro-inflammatory cytokines IL-1β and IL-18 is caspase-1 dependent. Understanding the role of these pro-inflammatory cytokines in the adjuvant activity of PCEP will provide critical information on how innate immunity influences the activation of adaptive immune responses.

## 5. Conclusions

In conclusion, our investigations reveal that the adjuvant PCEP activates the inflammasome resulting in secretion of IL-1β and IL-18, and this is a caspase-dependent process. However, PCEP had no effect on the expression of MHC class II or costimulatory molecules CD86 and CD40. In addition, PCEP directly activated B cells to produce IgM and also increased production of antigen-specific IFN-γ by T-cells. Thus, PCEP activates innate immunity, leading to increased antigen-specific T-cell responses.
